# Prevalence and Predictors of Use of Home Sphygmomanometers Among Hypertensive Patients

**DOI:** 10.7759/cureus.1155

**Published:** 2017-04-11

**Authors:** Hira Zahid, Aisha Amin, Emaan Amin, Summaiya Waheed, Ameema Asad, Ariba Faheem, Samreen Jawaid, Adila Afzal, Sarah Misbah, Kanza Majid

**Affiliations:** 1 Internal Medicine, Dow University of Health Sciences (DUHS), Karachi, Pakistan; 2 Internal Medicine, Jinnah Sindh Medical University (SMC); 3 Internal Medicine, Dow Medical College Karachi, Pakistan; 4 Dow Medical College Karachi, Pakistan

**Keywords:** home sphygmomanometers, hypertension, electronic arm and wrist devices, hbpm, blood pressure measurement

## Abstract

**Objective:**

Few studies have looked at the predictors of use of home sphygmomanometers among hypertensive patients in low-income countries such as Pakistan. Considering the importance of home blood pressure monitoring (HBPM), cross-sectional study was conducted to evaluate the prevalence and predictors of the usage of all kinds of HBPM devices.

**Method:**

This study was conducted in Karachi during the time period of January-February 2017. Adult patients previously diagnosed with hypertension visiting tertiary care hospitals were selected for the study. Interviews from the individuals were conducted after verbal consent using a pre-coded questionnaire. The data was analyzed using Statistical Package for the Social Sciences v. 23.0 (SPSS, IBM Corporation, NY, USA). Chi-squared test was applied as the primary statistical test.

**Results:**

More than half of the participants used a home sphygmomanometer (n=250, 61.7%). The age, level of education, family history of hypertension, compliance to drugs and blood pressure (BP) monitoring, few times a month at clinics were significant determinants of HBPM (P values < 0.001). It was found that more individuals owned a digital sphygmomanometer (n=128, 51.3%) as compared to a manual type (n=122, 48.8%). Moreover, avoiding BP measurement in a noisy environment was the most common precaution taken (n=117, 46.8%).

**Conclusion:**

The study showed that around 40% of the hypertensive individuals did not own a sphygmomanometer and less than 25% performed HBPM regularly. General awareness by healthcare professionals can be a possible factor which can increase HBPM.

## Introduction

Hypertension is one of the major risk factors for the development of cardiovascular diseases (CVDs) [1]. Elevated blood pressure (BP) causes almost half of the deaths due to stroke and ischemic heart diseases [1]. Although many treatment options are present, reaching a target BP is a common problem [2]. Accurate BP monitoring is vital for diagnosis and treatment of hypertension [3]. BP measurement is one of the most common tests done on a large number of people by usage of sphygmomanometer which is either manual or digital [4]. BP has been traditionally measured in the clinic setting but technologic advances have led to improvements in measuring clinic BP and allows for measuring BP outside the clinic [5]. Home blood pressure monitoring (HBPM) is gaining recognition as a vital tool for effective management of hypertension, allowing patients to actively participate in their treatment.

HBPM, according to a systematic review, increases patients’ adherence, provides effective BP control in hypertensive patients and evaluates antihypertensive medication efficacy [3]. In addition, the clinical effectiveness of HBPM is noted in guidelines for management of hypertension [6] and practice of HBPM [7-8]. Use of certified and accurate [9] sphygmomanometer in accordance with latest guidelines on how to measure BP is required for HBPM. Pakistan is a lower middle-income country with cardiovascular diseases (CVDs) being the second most common cause of mortality [10]. Hypertension being a risk factor for CVDs is widely prevalent in 55% of adults aged ≥ 18 years [11]. However, the ubiquity and predictors of use of the home sphygmomanometer, especially in low-income and lower-middle-income countries are understudied.

Thus, the primary objective of this was to determine the prevalence and predictors of the usage of all kinds of HBPM devices. The secondary objective was to investigate the influence of patient's characteristics, physician’s recommendation and frequency of BP measurement at clinics on how often patients assessed BP at home using sphygmomanometers.

## Materials and methods

This cross-sectional study was conducted in Karachi during the time period January-February 2017 after approval from the Institutional review board of Dow University of Health Sciences. Hypertensive patients who visited tertiary care hospitals were selected for the study. People who had secondary hypertension or were younger than 18 years of age were excluded from the sample. Four hundred and twenty-five patients in total were approached to take part in the study, out of which 15 (3.5%) refused to give consent. Of the remaining 410 patients, five (1.2%) left the interview incomplete. Therefore, the final sample size was 405, with a co-operation rate of 95.3%.

A pre-coded questionnaire, which was available in both English and Urdu (the Pakistani national language) was used to conduct all interviews. This was done to allow the participants to complete the survey in the language they were more comfortable with using. The questions were carefully worded to reduce any difficulty in comprehension. The questionnaire was studied and approved by two research analysts. All interviewers chosen to conduct the interviews were fluent in both languages in order to effectively deal with any queries that the participants had. Verbal consent of participants was taken. Interview bias was reduced by random sampling and interviewers were previously briefed to maintain uniformity in procedures. Recall bias was eliminated by asking recent and relevant information in the questionnaire. Imputation was avoided during this study. Standard medical terms were also defined to eliminate ambiguity. A sphygmomanometer was defined as ‘an instrument used for measuring BP’ and hypertensive individuals were defined as ‘patients diagnosed with high BP (140/90 mm of Hg on more than two separate occasions) previously by a doctor’.

The questionnaire was divided into three sections. In the first section, details regarding their age, gender, educational status, co-morbidities and normal range of BP were asked. The BP readings were then divided into four categories of ranges using standard guidelines [6]: controlled systolic BP, controlled diastolic BP, uncontrolled systolic BP, and uncontrolled diastolic BP. The prevalence of home sphygmomanometers was also investigated in this section. The second section inquired the possible predictors of owning a sphygmomanometer, such as the patients’ lifestyle practices, the history of hypertension in their family and their opinions as to whether they believed the use of a home sphygmomanometer to be satisfactory and effective. The influence of physicians’ recommendations and how frequently the patients visited clinics to get their BP assessed was also noted. The final section tackled the basic practices of people who performed HBPM and the nature of the devices they owned.

The data collected in the study were expressed in terms of frequencies and percentages for categorical responses and was analyzed using Statistical Analysis for Social Science (SPSS) v. 23.0. (SPSS, IBM Corporation, NY, USA) Chi Square test was applied to evaluate the predictors of sphygmomanometer ownership among hypertensive patients. A P value of less than 0.05 was regarded to be significant.

## Results

The mean age of the sample was 50.5 years and more than half of the sample comprised of female participants (n=213, 52.6%). Around half of the patients had pursued a tertiary level of education (n=188) while 25.8% (n=90) had received a secondary level of education. Fifty-one individuals were smokers (12.6%) while a vast majority of patients reported having a family history of hypertension (n=254, 62.7%) and comorbidities associated with the disease (n=306, 75.6%).

**Figure 1:**
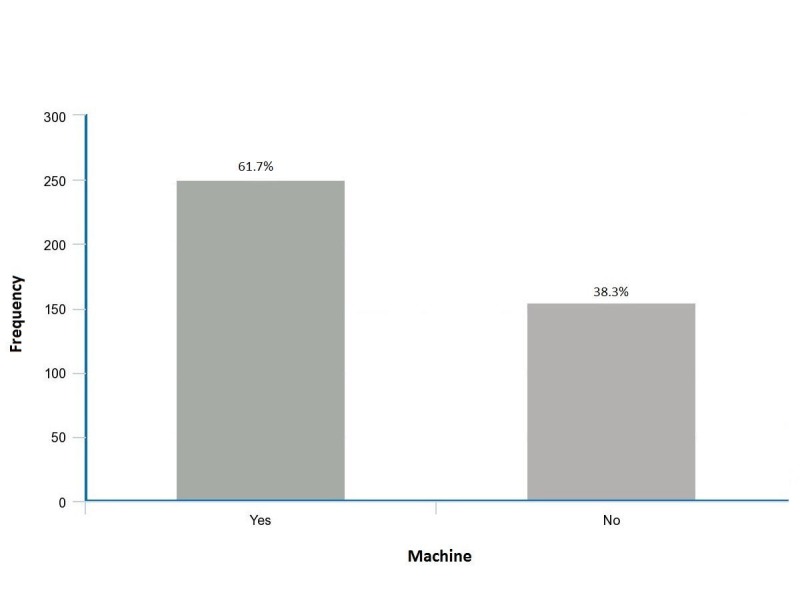
Shows the prevalence of sphygmomanometers among the sample population

The majority of the participants (n=250, 61.7%) owned a sphygmomanometer (Figure 1). Of the total participants, those who were between the age of 40 and 59 years (n=246, 60.7%) had a statistically higher chance of owning a sphygmomanometer (P=0.002). People who did not exercise were also more likely to own the device (P <0.001). Those individuals who had hypertensive patients in their immediate family (n=253, 62.5%) performed HBPM, as compared to those with no family history of hypertension (P=0.005). Sleeping patterns had a significant correlation with owning a sphygmomanometer (P=0.004). People who got between six to eight hours of sleep each night (n=232, 57.3%) were more likely to keep a sphygmomanometer at home. Gender (P=0.262), cigarette smoking (P=0.438) and the presence of any associated comorbidities, such as previous myocardial infarction, hyperlipidemia, and stroke (P=0.833), were found to have no significant impact on the usage of sphygmomanometers. There was, however, a statistical significance (P <0.001) for the relation between the level of education and the ownership of a sphygmomanometer, whereby individuals with a tertiary level of education (n=188, 46.4%) were more likely to practice HBPM (Table 1).

**Table 1:** Compares characteristics of people who owned a sphygmomanometer with those who did not own a sphygmomanometer

		SPHYGMOMANOMETER OWNERSHIP		p-value
		YES	NO	
Gender	Male	124 (49.6%)	68 (43.9%)	0.262
	Female	126 (50.4%)	87 (56.1%)	
Age	18-39 years	41 (16.5%)	34 (22.2%)	0.002
	40-59 years	144 (57.8%)	102 (66.7%)	
	60 years & above	64 (25.7%)	17 (11.1%)	
Education	Primary	13 (5.8%)	33 (26.2%)	<0.001
	Secondary	52 (23.3%)	38 (30.2%)	
	Tertiary	149 (66.8%)	39 (31.0%)	
	None	9 (4.0%)	16 (12.7%)	
How busy do you find your job to be?	Very busy	69 (30.0%)	52 (36.1%)	0.465
	Moderate busy	103 (44.8%)	58 (40.3%)	
	Not busy	58 (25.2%)	34 (23.6%)	
Do other members of your family have hypertension?	Yes	169 (68.0%)	84 (54.2%)	0.005
	No	80 (32.0%)	71 (45.8%)	
Do you exercise?	Yes	89 (35.6%)	30 (19.4%)	<0.001
	No	160 (64.4%)	125 (80.6%)	
Do you smoke?	Yes	34 (13.6%)	17 (11.0%)	0.438
	No	216 (86.4%)	138 (89.0%)	
How many hours do you sleep?	<6 hours	68 (27.3%)	49 (31.6%)	0.004
	6-8 hours	136 (54.6%)	96 (61.9%)	
	>8 hours	45 (18.1%)	10 (6.5%)	
Do you take your medications regularly and on time?	Yes	209 (83.6%)	104 (63.1%)	<0.001
	No	41 (16.4%)	51 (32.9%)	
Were you recommended to use a home sphygmomanometer?	Yes	165 (66.0%)	30 (19.4%)	<0.001
	No	85(34.0%)	125 (80.6%)	
Who recommended you to use a home sphygmomanometer?	Doctor	104 (61.9%)	19 (70.4%)	0.677
	Family/Friends	58 (34.5%)	7 (25.9%)	
	Advertisements	6 (3.6%)	1 (3.7%)	
How effective/useful do you think is the presence of a sphygmomanometer at your home?	Not at all	8 (3.2%)	24 (15.5%)	<0.001
	A lot	242 (96.8%)	131 (84.5%)	
How satisfied are you with measuring your BP at home?	Satisfied	232 (92.8%)	111 (71.6%)	<0.001
	Not so satisfied	18 (7.2%)	44 (28.4%)	
How would you rate your BP?	In Control	131 (52.6%)	72 (48.6%)	0.445
	Out of Control	118 (47.4%)	76 (51.4%)	
	Yes	188 (75.2%)	118 (76.1%)	
Do you have any comorbidities (eg. previous MI, hyperlipidemia, stroke etc)?	No	62 (24.8%)	37 (23.9%)	0.833
	Uncontrolled	107 (44.4%)	75 (55.1%)	
Systolic BP	Controlled	134 (55.6%)	61 (44.9%)	0.045
	Uncontrolled	141 (58.8%)	91 (74.0%)	
Diastolic BP	Controlled	99 (41.3%)	32 (26.0%)	0.004
	>Once a day	1 (0.4%)	2 (1.4%)	
How frequently do you get your BP checked by a doctor or nurse?	Once a day	13 (5.3%)	19 (13.0%)	<0.001
	Few times a week	12 (4.9%)	21 (14.4%)	
	Once a week	16 (6.5%)	13 (8.9%)	
	Few times a month	129 (52.2%)	73 (50.0%)	
	Rare/When needed	76 (30.8%)	18 (12.3%)	

The majority of the participants who took their medications on time (n=313, 77.3%) also owned a sphygmomanometer (P= <0.001). More than half of the patients did not receive any recommendation to use a home sphygmomanometer (n=210, 51.9%). Those who were recommended, however, had a significantly higher chance of owning the device (P= <0.001). By whom the recommendation was made was not a significant predictor (P=0.677). Individuals who got their BP assessed few times a month by a doctor or nurse (n=202, 49.9%) were more likely to keep a sphygmomanometer at home (P= <0.001). The participants’ own opinion of whether their BP was under control or not was found to be insignificant to the prevalence of sphygmomanometers (P=0.445). However, patients with a controlled systolic BP (P=0.045) and an uncontrolled diastolic BP (P=0.004) had a higher likelihood of owning a sphygmomanometer. Almost all (n=242) of the people who owned a sphygmomanometer found its usage very effective. Most people felt satisfied with measuring their BP at home (n=343, 84.7%) (Table 1).

Table 2 outlines the practices of sphygmomanometer owners. Most of the participants stated that they assessed their BP few times a month (n=76, 30.4%), whereas 9.2% (n=23) only assessed it when feeling ill. It also shows that out of all the participants who owned a sphygmomanometer, 48.8% (n=122) owned the manual type. For the digital type, 56.0% (n=140) of the participants did not calibrate it, while a majority of 70.0% (n=175) preferred wearing it on their arm instead of their wrist (n=64, 25.6%) or other any area (n=11, 4.4%). Out of all the precautions listed, most of the participants (n=117, 46.8%) avoided measuring their BP in a noisy environment, while the second most common precaution was to avoid measuring BP right after taking a meal (n=92, 36.8%). Around one-third (n=83) of the sphygmomanometer, owners reported taking no precautions while measuring their BP at home. Most of the patients stated that their readings at home sometimes matched the readings taken in a clinical setting (n=123, 49.2%) and a majority of the sample (n=209, 83.6%) stated that their readings were not higher when checked by a doctor. More than half of sphygmomanometer users agreed that they do not repeat or take an average of their readings (n=134, 53.6%) and a vast majority of the people did not measure their BP at a fixed time during the day (n=198, 79.2%).

**Table 2:** Shows the frequency of practices of sphygmomanometer owners

	Frequency
Type of sphygmomanometer	
Manual	122 (48.8%)
Digital	128 (51.2%)
Do you recalibrate it?	
Yes	110 (44.0%)
No	140 (56.0%)
Where do you wear it?	
Arm	175 (70.0%)
Wrist	64 (25.6%)
Other	11 (4.4%)
Do you take any precautions when measuring your BP?	
After having a big meal	92 (36.8%)
Within 30 minutes of caffeine intake	53 (21.2%)
In a noisy environment	117 (46.8%)
No precautions	83 (33.2%)
Does your BP reading at home match with your BP taken at a clinic?	
Always	102 (40.8%)
Sometimes	123 (49.2%)
Rarely	25 (10.0%)
Is your BP higher when measured by a doctor as compared to when measured at home?	
Yes	41 (16.4%)
No	209 (83.6%)
Do you ever repeat your readings and take average?	
Yes	116 (46.4%)
No	134 (53.6%)
What time of the day do you measure your BP?	
After waking up	18 (7.2%)
Before taking a meal	8 (3.2%)
After taking a meal	7 (2.8%)
Before going to bed	19 (7.6%)
No specific time	198 (79.2%)
How frequently do you assess your BP?	
Two or more times a day	22(8.8%)
Once a day	36 (14.4 %)
Few times a week	445 (18.0%)
Once a week	8 (19.2%)
Few times a month	76 (30.4%)
Only when feeling ill	23 (9.2%)

## Discussion

The study shows that 61.7% of the sample population used a sphygmomanometer yet less than 25% monitored their BP regularly. Worldwide surveys show that the use of BP monitoring devices varies from 30-70% [12-14]. As shown by a multi-centre survey stating, 640 (75%) out of 855 hypertensive patients in Italy [15] used home sphygmomanometers. However, the prevalence of home sphygmomanometers in UK and Canada is around 30% [16] and 45.9% [12] respectively. This is surprising because compared to other developed countries; Pakistan is a developing country with a lower income and an inadequate education. Benefits of HBPM are stated in worldwide literature and include better patient compliance and prognosis ultimately leading to therapeutic inertia.

Previous studies also show groups of healthcare professionals playing an effective role in the improvement of self-management of BP [17-18]. About 90–95% of the physicians in Japan recommend the management of high BP [19] and a vast majority of hypertensive patients in the United States and Japan have a home sphygmomanometer [8,19], indicating the importance of physician recommendations and patient counseling in its usage. Therefore it is not surprising that about 66% of hypertensive patients were recommended to use home sphygmomanometer out of which 61.9% of the recommendations were made by their respective doctors. In addition, the results indicate that the level of education had a great impact on the use of home sphygmomanometers. People with secondary and primary education tended to make less use of home sphygmomanometers in comparison with people with a tertiary education. This is due to a lack of awareness on the importance of HBPM and its consequences, amongst participants with lower levels of education. Hence they were not exposed to health literacy from where they could draw out messages about healthy lifestyle to prevent and manage diseases in future and comprehend health messages when confronted with one [20]. Ergo, they remained uninvolved in their own treatment and objectively relied on the doctor, who was then given the divine status, especially in Pakistani culture. In contrast, the well-educated participants were seen to be active in the prevention of hypertension or self-assessment of it by HBPM. Also, people with less education often face greater socioeconomic stresses including occupations offering lower salaries, where the cost of a sphygmomanometer could have been a discouraging factor as stated by Akpolat, et al. [21].

According to a nationwide screening program, the prevalence of hypertension doubled with a positive family history [22]. Familial hypertension is a major predictor of the use of home sphygmomanometers in this study where the greatest percentage (68%) of the users had a hypertensive family member. This implies a positive correlation between participants with hypertensive family members and awareness of the importance of HBPM. Also, since individuals with a family history of hypertension are at a greater risk of acquiring it, they are more likely to be cautious about the risk factors and to monitor their BP on a regular basis for personal satisfaction (42%).

It was found that middle-aged participants, who did not exercise in leisure time, did not smoke, had moderately busy jobs, slept for six-eight hours but had a family history of hypertension made the majority of home sphygmomanometer users. This is mainly because of precautionary attempts made by such participants due to the fear of association between increasing age [23] and hereditary factors with hypertension [24]. In Pakistan, usually, there are one-two financial providers per family with their retirement age up to 60 years. Hence being in the middle ages, the participants are occupied with their job activities which are inversely related to leisure physical activities [25]. In a busy lifestyle, it is only convenient to check one’s BP constantly to attain early diagnosis of hypertension. These participants were adherent to their medications and were not involved in unhealthy habits like smoking which further suggests that they were health conscious but due to their overburdened life, they could not adopt to an active lifestyle. However, most of the participants older than 60 years did not own a home sphygmomanometer possibly because of negligence, indolence, forgetfulness and sometimes even a deteriorating will to live. In addition, bearing to complexities of devices like home sphygmomanometer also decreases amongst such participants.

The guidelines for home sphygmomanometers dictate the need for calibration of the device at least once a year [26] and the repetitive measure of the BP [25]. Yet more than half of the participants neither recalibrated their devices nor averaged out their readings which could also explain why a notable number of people (46%) stated that sometimes their readings taken at home did not match with those taken at a clinic. In addition, most of the participants checked their BP once a month when the guidelines instruct that home BP should be measured twice a day [27]. Hence, there is avoidable clinically significant over- and under-detection of hypertension as mentioned above. This striking finding could have been because of the poor standard of training of home sphygmomanometer users as to how to use the device. Even though the characteristics of such training were not questioned in the survey, the common mistake made by most of the users aided us to make such a claim. This situation is worrisome because most of the people who used home sphygmomanometer defied the purpose of HBPM which is to keep a close track of one’s BP. However, this can be reverted if the users take their devices with them to the physician’s clinic while their visit. However, 69% of the people used their arms instead of wrists to monitor BP as recommended by two specific guidelines [7-8] which could be because most local clinicians check BP on their patient's arms and people tend to replicate their physician's gestures.

The majority of the participants who were frequent users of home sphygmomanometer still had isolated systolic hypertension. This could be due to incomplete knowledge, haphazard routines and unawareness of the proper techniques related to home sphygmomanometers that address measurement standardization and frequency, checking and documenting of the pulse rate and pressure. Hence, their BP measurements might have some errors. To avoid this in the future, proper HBPM protocol should be provided by the physicians, sellers, and even skilled professional staff so that the purpose of HBPM can be fulfilled. According to Tyson, et al. [28], training increased the ownership of home sphygmomanometers by 64.8%. Therefore, emphasis on strategic education of the population along awareness programs regarding HBPM is greatly beneficial, especially for the ones with hypertension or a CVD. In addition, the accessibility of home sphygmomanometers in terms of cost and motivational emphasis by physicians to ensure adherence and self-management of hypertension should be promoted.

There are several limitations in this study which need to be considered. First, the sample size was relatively small; however, it represented a population of varied socioeconomic levels. Second, the comparison between the performance of trained and untrained users was not studied. Third, the Hawthorne effect which increased the propensity of purchasing a home sphygmomanometer and rounding off the BP to the commonly known standard value of 120/80 mm of Hg was not excluded. Fourth, some important information like the brand, model, and price of home sphygmomanometers was missing. In addition, the cuff size of the upper-arm device used and the consistency of the arm circumference of the patient was not considered. Obesity being common in hypertensive individuals results in a larger mean arm circumference [29] and to obtain an accurate reading, a suitable cuff size is very important. The future researchers should exploit the relation between obesity, socio-economic classes, and HBPM, along with the detailed study of the characteristics of the home sphygmomanometers.

## Conclusions

More than half of the hypertensive patients in this study owned a home sphygmomanometer. However, possibly due to a lack of instruction for recalibration and the importance of repeated measurements by healthcare professionals, HBPM may be unreliable. Therefore, it is essential that a proper plan is developed in order to educate and guide the users about the proper procedure of HBPM. In addition, age, education, lifestyle and familial hypertension vastly affected the use of home sphygmomanometer. General awareness about home sphygmomanometer can motivate the patients to perform HBPM to ensure self-management of hypertension.
